# Enhancing plant growth promoting rhizobacterial activities through consortium exposure: A review

**DOI:** 10.3389/fbioe.2023.1099999

**Published:** 2023-02-10

**Authors:** Anamika Singh, Virendra Kumar Yadav, Rajendra Singh Chundawat, Raya Soltane, Nasser S. Awwad, Hala A. Ibrahium, Krishna Kumar Yadav, Simona Ioana Vicas

**Affiliations:** ^1^ Department of Biosciences, School of Liberal Arts and Sciences, Mody University of Science and Technology, Sikar, Rajasthan, India; ^2^ Department of Basic Sciences, Adham University College, Umm Al-Qura University, Makkah, Saudi Arabia; ^3^ Chemistry Department, Faculty of Science, King Khalid University, Abha, Saudi Arabia; ^4^ Biology Department, Faculty of Science, King Khalid University, Abha, Saudi Arabia; ^5^ Department of Semi Pilot Plant, Nuclear Materials Authority, El Maadi, Egypt; ^6^ Faculty of Science and Technology, Madhyanchal Professional University, Bhopal, India; ^7^ Department of Food Engineering, University of Oradea, Oradea, Romania

**Keywords:** rhizobacteria, rhizosphere, synergistic relation, consortium, sustainable development, cross-feeding

## Abstract

Plant Growth Promoting Rhizobacteria (PGPR) has gained immense importance in the last decade due to its in-depth study and the role of the rhizosphere as an ecological unit in the biosphere. A putative PGPR is considered PGPR only when it may have a positive impact on the plant after inoculation. From the various pieces of literature, it has been found that these bacteria improve the growth of plants and their products through their plant growth-promoting activities. A microbial consortium has a positive effect on plant growth-promoting (PGP) activities evident by the literature. In the natural ecosystem, rhizobacteria interact synergistically and antagonistically with each other in the form of a consortium, but in a natural consortium, there are various oscillating environmental conditions that affect the potential mechanism of the consortium. For the sustainable development of our ecological environment, it is our utmost necessity to maintain the stability of the rhizobacterial consortium in fluctuating environmental conditions. In the last decade, various studies have been conducted to design synthetic rhizobacterial consortium that helps to integrate cross-feeding over microbial strains and reveal their social interactions. In this review, the authors have emphasized covering all the studies on designing synthetic rhizobacterial consortiums, their strategies, mechanism, and their application in the field of environmental ecology and biotechnology.

## 1 Introduction

Microorganisms are omnipresent in nature and are considered to be present with every living organism on the earth (Kirchman, 2018). From the vast literature, it has been found that microorganisms are present in all the parts of a plant for instance in the phylloplane areas, phyllosphere, or in the rhizosphere, the first two are mainly involved with the activities on the external surface of the plant and in severe cases may have a role in the plant diseases (Saeed et al., 2021). While the rhizospheric microbial species are of utmost importance due to their direct role in plant growth, health, and nutrition. In the last decade, rhizosphere-based microbial studies have gained huge attention among the scientific communities. The rhizosphere is an area where there are high biological and chemical activities occurs. In this zone, plant exudates are released which are used by the microbes as a source of energy (Prashar et al., 2013). The interaction of plants with microbes in the rhizospheric region helps in maintaining the soil fertility and health of the plant (Kumar et al., 2019a). One such group of bacteria which is present in the rhizosphere is planted growth-promoting bacteria (PGPR).

PGPR are free-living bacteria that have a direct role in the growth of the plant and rooting system among the plants (Hayat et al., 2010). It has also a role in nitrogen fixation, solubilization of insoluble phosphates, and secretion of several plant hormones. Due to the importance of PGPR in the ecosystem, it has drawn the attention of scientists in the last few years. Today PGPR is widely used in the field of agriculture, especially in biofertilizers, biogeochemical cycling of minerals, etc., (Shah et al., 2021a). From the pieces of literature, it has been found that only 2%–5% of rhizosphere bacteria are PGPR and they have been considered an important tool for sustainable agriculture (Antoun and Prevost (2005). From the various studies, it has been found that PGPR is adsorbed on the surface of the soil by the ion exchange process. Most plants are notable to use organic sources of elements so, these PGPR have a role in providing the plants with the inorganic form of elements. This maintains the fertility of the soil and is an important aspect of sustainable agriculture (Goswami et al., 2016). The PGPR bacteria have mainly members from the genera like *Arthrobacter, Beijerinckia, Burkholderia, Derxia, Acetobacter, Acinetobacter, Klebsiella, Ochrobactrum, Bacillus, Enterobacter, Gluconacetobacter, Pantoae, Alcaligenes, Arthrobacter, Pseudomonas, Rhodococcus, Serratia, Azoarcus, Azospirillum, Azotobacter, Herbaspirillum, Stenotrophomonas, Lactobacillus, Paenobacillus, and Zoogloea* (Vega-Celedón et al., 2021).

In the last few years, the trend toward PGPR studies has shown the role of microbial consortium in PGP activities. There are several reports where a vast variety of microorganisms are found in their hostile environment where they are interacting with other microorganisms intra and interspecifically. In the natural environment, 99% of microorganisms are found in the form of a microbial consortium (Dong et al., 2017). Till the 20th century, there are enormous studies that have shown that single microorganisms can play a beneficial role in plant growth, but in nature, it is noticeable that multiple species in microbial consortia can perform several beneficial functions for our ecosystem than a single microorganism. Interaction between PGPR and plants is synergistically driving benefits for the plant microbiome (Santos and Olivares 2021). Plants also promote PGPR growth through the production of various storage substances and also root exudates, which are used by PGPR for nutrition (Rahina et al., 2018). This tool is used to establish a symbiotic association between plants and PGPRs like mycorrhizal nitrogen-fixing root nodulation (Siddiqui et al., 2021) and this establishment can be endophytic or exophytic (Govindasamy et al., 2019). Once the association is established, PGPR shows benefits for plant growth, health, and traits through their direct and indirect mechanism, (Neshat et al., 2022). There are several reports which describe the role of monoculture in the case of PGPR but very few attempts were made to enhance the PGP activities by using microbial consortium. Some of the recent works done in this area are described below.

Fatima et al., 2022 reported the isolation of multifaced PGPR, from natural suppressive soils. Moreover, the investigators also studied the potential of such multifaced PGPR for bio-control activities and growth-promoting potential against the etiological agent of chickpea wilt (Fatima et al., 2022).

Recently a group of scientists reported the beneficial role of PGPR the in the remediation of nitrile pollutants from the cropland in order to enhance crop productivity. The investigators concluded that both nitrile and cyanide have a negative impact on plant health. Further investigators concluded that nitrile pollution can be managed by using nitrilase enzyme along with PGPR. The authors also suggested that the breakdown of nitrile by such activities will lead to the formation of nitrogen which could be utilized by the plant (Vaishnav et al., 2022).

Abbasi et al., 2022 developed a consortium of *Streptomyces* species and used them for the bell pepper fruits under controlled greenhouse conditions. The investigators reported that there was improved quality of bell pepper fruits, induced plant defencepriming, and moreover, the team led by Abbasi et al. (2022) reported that the *Streptomyces* consortium changed the microbial communities of the rhizosphere.

Samain et al., 2022 developed a PGPR microbial consortium using Paenibacillus sp. strain B2 and Arthrobacter sp. strain AA. The developed consortium was used for Zymoseptoria tritici and drought stress. The investigators reported that the developed consortium provided effective and durable systemic wheat-induced resistance (Samain et al., 2022).

By searching PGPR, bacterial consortium on science direct it was found that very less work is done in this area in comparison to other research activities. For instance, on searching PGPR, bacterial consortium, during the years 2017–2022, authors found that in 2017 about27 articles were published in 2018 about 34 articles, 65 articles in 2019, 95 articles in 2020, 126 in 2021, and 178 to date in 2022. The trend suggests that there is a gradual increase in PGPR-based research activities in the scientific community. In these last six years about a maximum were research articles i.e., 194, followed by book chapters i.e., 170, and review articles i.e., 108, the remaining were encyclopedias and other reports.

For the last six years researchers are working on designing rhizobacterial consortium for enhancing plant growth, their yield of production, soil health, as a biocontrol agent, and stabilizing the rhizobacterial interactions (Kamalnath et al., 2019; Swandi et al., 2019; Asyiah et al., 2020; Kumar et al., 2020; Shang and Liu 2020; Vaid et al., 2020; Redondo-Gómez et al., 2021; Karuppiah et al., 2022; Shabaan et al., 2022) In the current review, the authors have emphasized the role of rhizosphere microbes in PGP activities. Moreover, the authors have also highlighted the recent trends in PGPR-based research works. In addition to this, authors have emphasized the enhancement of the PGPR activities by using microbial consortium. The authors have emphasized the application of PGPR in environmental ecology and biotechnology. Such type of practices will lead to sustainable agriculture-led production of crops.

## 2 PGPR mechanisms and their beneficial activities

**FIGURE 1:**
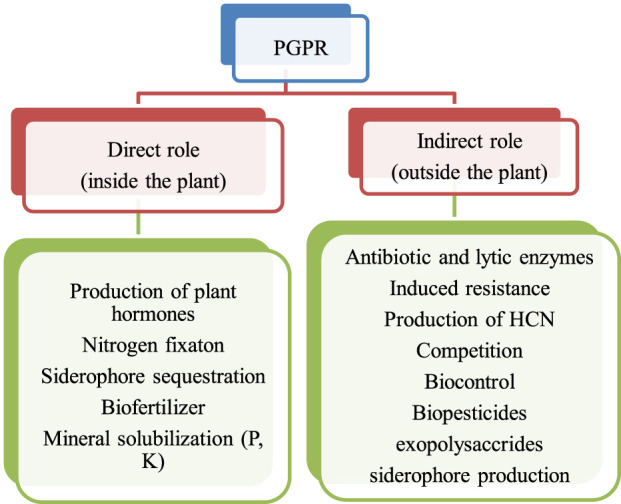
Types of PGPR activities in the rhizosphere (Kundan et al., 2015; Nath et al., 2018).

From the various pieces of literature, it has been found that there are basically two types of mechanisms associated with the beneficial effect of PGPR activities in the rhizosphere. For instance, it is the direct type and indirect type are shown below in Figure 1. Both the mechanism of PGPR activities is important and leads to sustainable agricultural crop production (Ahemad and Kibret 2014).

**FIGURE 2:**
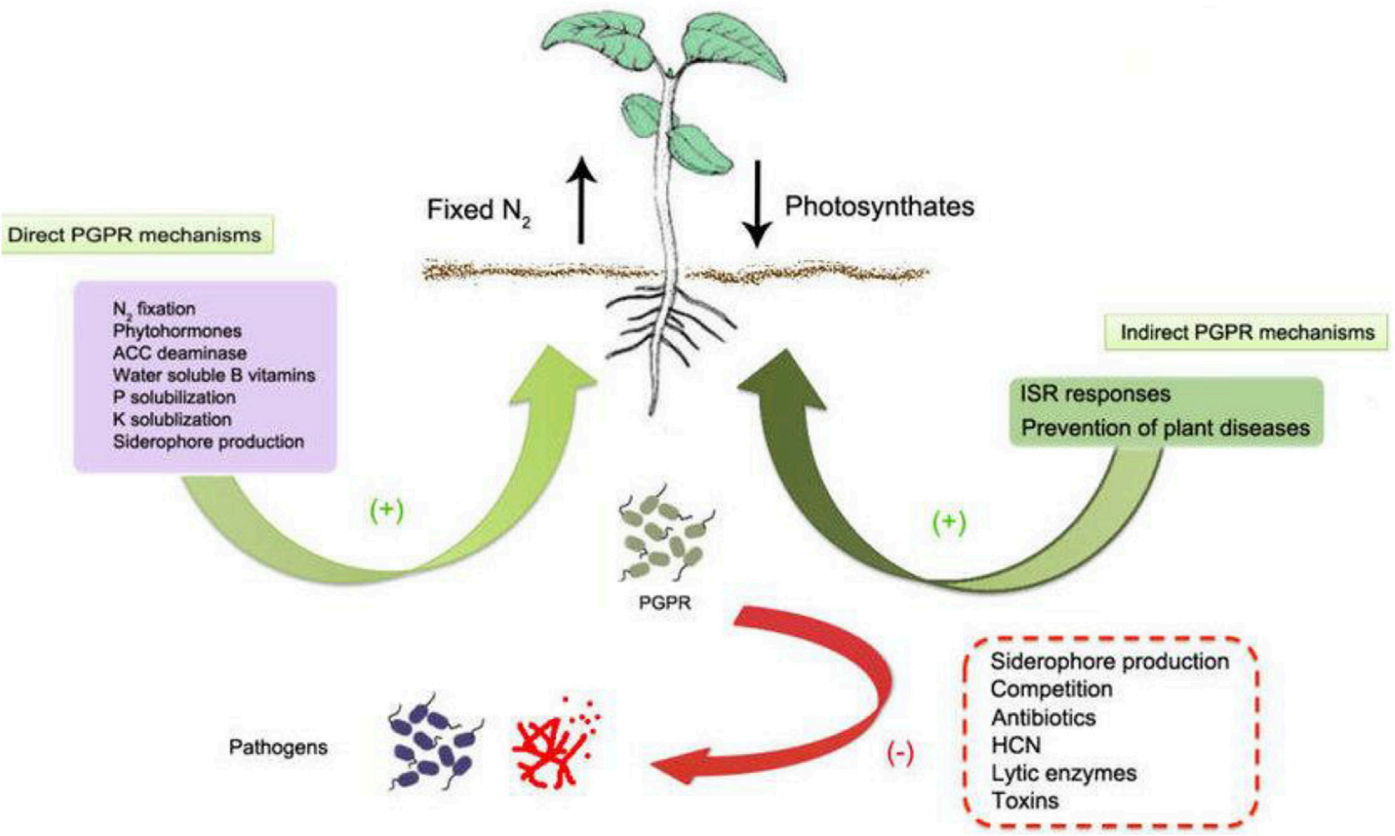
PGPR mechanism in the rhizosphere adopted from (Kamili et al., 2019; Nazir and Kamili, 2018).

**FIGURE 3:**
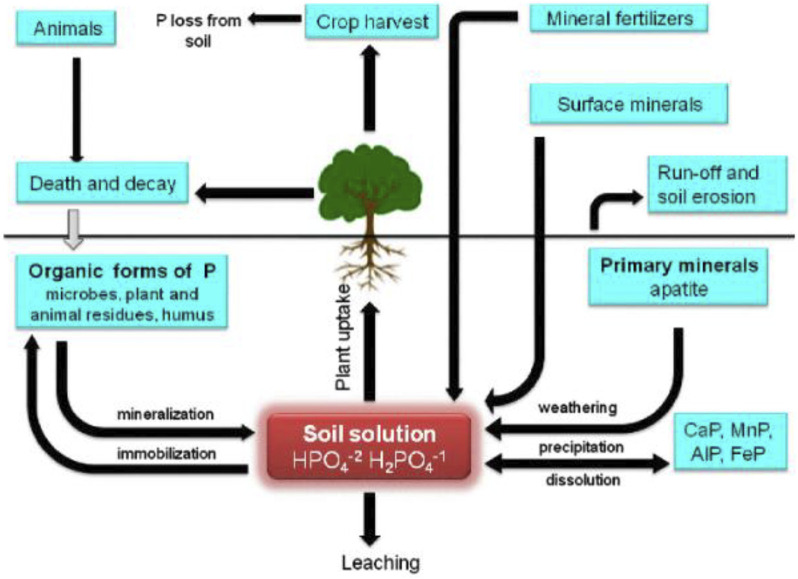
Movement of phosphorus in soil systems adopted from (Ahemad and Kibret 2014).

**FIGURE 4:**
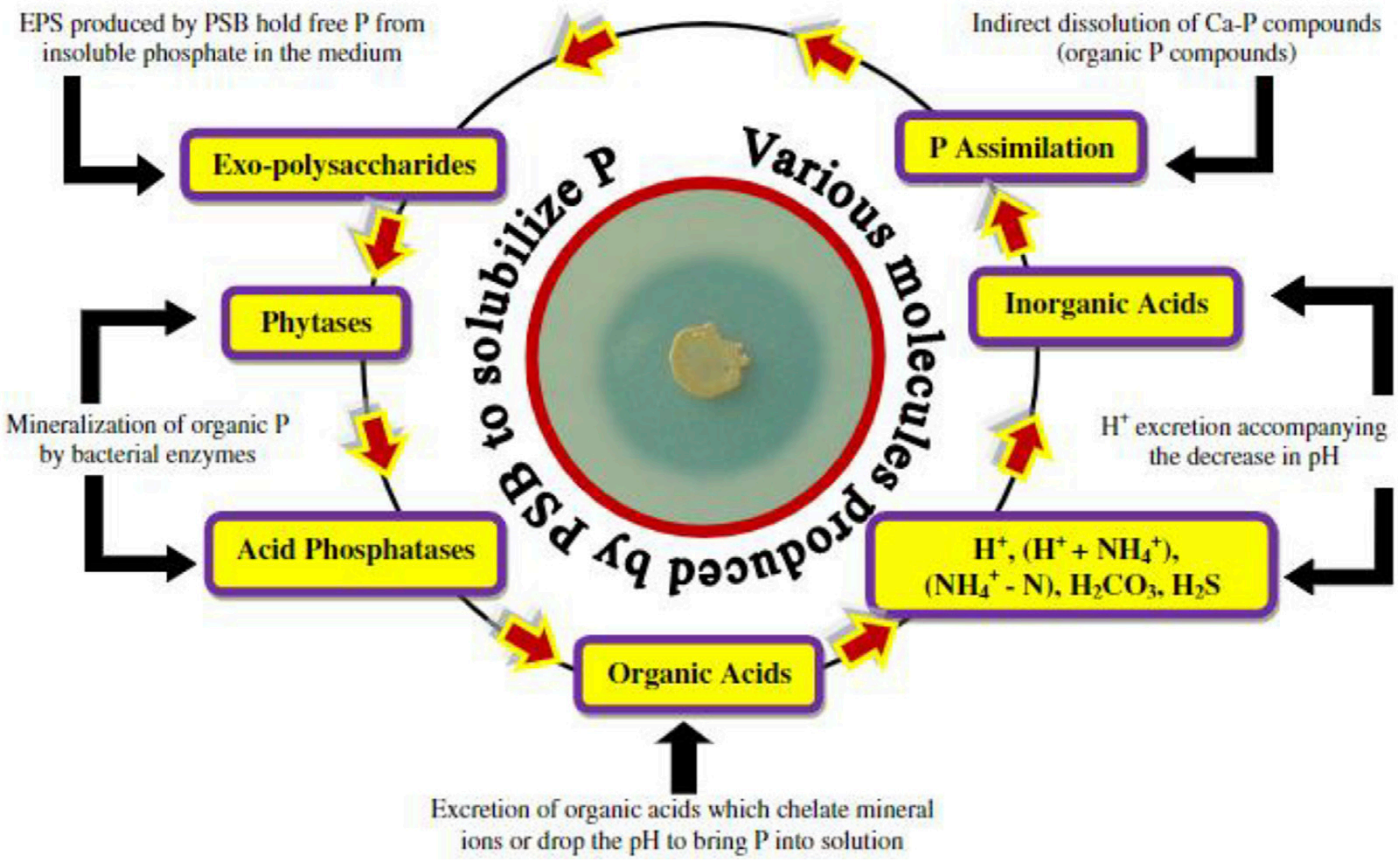
Different organic and inorganic substances produced by phosphate solubilizing bacteria for P, solubilization from soil adopted from (Ahemad and Kibret 2014).

In direct mechanism, PGPR promotes plant growth by releasing various useful substances like phosphates, potassium, silicon, and zinc and the uptake of biologically fixed nitrogen, chelation of iron and other micronutrients and increase in available geospheric oxygen that stimulates aeriform biomass production, root development and also in stem elongation (Constantia and Ferniah 2020). PGPR also synthesizes phytohormones, such as gibberellins, auxins, indoleacetic acid, cytokinin, abscisic acid, and ethylene (Patel and Saraf 2017). The production of an enzyme 1-aminocyclopropane 1-carboxylate deaminase (ACC) reduces the level of ethylene in the roots of crops, therefore boosting the density and length of the roots (Singh and Jha 2017). Figure 2 shows the PGPR mechanism in the rhizosphere, while Figure 3 shows the movement of P in the soil. Figure 4 shows the various substances produced by phosphate-solubilizing bacteria (PSB), in the soil.

In an indirect mechanism, PGPR is inducing systemic resistance and stimulates the innate resilience of the plant, and involves modification of the rhizosphere ecology (Adrees et al., 2019). PGPR release various substances that activate the protection mechanism of plants against various pathogenic microorganism and also promote the synthesis of physical and chemical barriers against abiotic stress, these substances are siderophore, pigments, antibiotics, organic acids, water-soluble vitamins, and different volatile organic compounds like monoterpene alcohols (Chandran et al., 2021). In return, plants allow PGPR to become more competitive in their niche colonization so that they can make interference with the quorum detection signal (Hartmann 2020) and also enables them to inhibit the formation of biofilms by pathogenic bacteria (Uruén et al., 2020). PGPR is also able to remediate contaminated soils (Vaishnav et al., 2022).

Because of their multifunctional benefits, PGPR is in leading demand in agroforestry management (Phale 2018). PGPR are key ecosystem service providers, they drive multifunctional processes and carry interaction with differential microbial communities (Lindemann 2019). This complex interconnected network of PGPRs responsible to maintain the transportation and circulation of energy and recirculation of resources to an entire ecosystem that affects soil microfauna and vegetational biome (Sylia et al., 2022).

### 2.1 Major mechanism of PGPR

Although both direct and indirect mechanism of PGPR equally provides benefits for plants’ health nitrogen fixation is the major mechanism of all PGPR (Mahmud et al., 2020), because all rhizobacteria have the capacity to fix atmospheric nitrogen, hence called rhizobacteria and there is the likelihood that each of the direct and indirect mechanism may present or absent in all rhizobacteria.

Most of the proteins, signaling molecules carries nitrogen as a key component. Nitrogen is also one of the crucial macronutrients for plant growth. Although nitrogen fertilizers are very costly in agricultural systems, thus affect the production of many crops. Exhaustive use of synthetic nitrogenous fertilizers in modern agriculture causes harmful effects on our natural environmental system. To preserve the health of the environment, PGPR is the resource of nitrogen and use as a natural biofertilizer to accomplish the requirement of nitrogen and other nutrients for plants without harming their productivity and the health of the environment (Monterio et al., 2021). Nitrogen is prominently present in the atmosphere in a gaseous form which is impassable to plants and animals. Assimilation of nitrogen by plants, atmospheric nitrogen is required to convert into ammonia, and this conversion is driven by nitrogenase enzymatic complex containing nitrogen-fixing microorganisms and the process called biological nitrogen fixation (Li et al., 2021). Rhizobia is the universal group of bacteria to fix nitrogen which is abundantly present in the rhizospheric area of soil (Shimonda et al., 2020). For sustainable production of crop production PGPR are the best source to provide nitrogen. Some examples of nitrogen-fixing microorganisms are, *Actinobacteria, Micrococcales, Microbacteriaceae*, *Proteobacteria, Alphaproteobacteria, Rhodospirillales, Acetobacteraceae, Roseomonas* (Zhang M. et al., 2020) *Azotobacter vinelandii*, Azospirillum*zeae*, *Acetobacter diazotrophicus*, *Burkholderia tropica* and *Achromobacter insolitus* etc., (Singh et al., 2020).

There are various examples where plant growth-promoting compounds are released by PGPR which are shown below in Table 1.

**TABLE 1:** Growth-promoting substances released by PGPRs.

PGPR	Plant growth-promoting characters	References
Pseudomonas sp.	IAA, siderophores, HCN, NH_3_, exo-polysaccharides (EPS), PO_4_^3-^solubilization	(Fazeli-Nasab & Sayyed, 2019)
*Agrobacterium radiobacter*	Antibiotics	(Mohanram and Kumar, 2019)
*Pseudomonassp*	IAA	(Jaleel et al., 2021)
*Pseudomonas aeruginosa*	PO_4_^3−^solubilisation, ACC deaminase	(Linu et al., 2019)
*Paenibacillusxylanexedens*	Chitinase production	(Hussin and Ab Majid, 2020)
Bacillus species PSB10	IAA, siderophores, HCN, NH_3_	(Ahemad and Kibret, 2014)
*Stenotrophomonas rhizophila*	Amylase synthesis	(Singh and Jha, 2017)
*Klebsiella pneumonia*	N_2_ fixation	(Sharma et al., 2022)
*Azotobacter chroococcum*	Gibberellin	(Zhang et al., 2019a)
Bacillus subtilis Rhizo SF48	ACC deaminase	(Gowtham et al., 2020)
*Azotobacter, Bacillus, Pseudomonas, Rhizobium*	Phytohormone production	(Baber et al., 2018)
*Bacillus spp., Burkholderia spp., Pseudomonas*	Macronutrients production	(Baber et al., 2018)
*Bacillus and Pseudomonas species*	Lytic enzyme production	(Mabood et al., 2014)
*Pseudomonas, Bacillus, Burkholderia, Agrobacterium, Paenibacilluspolymyxa, Xanthomonas*	Volatile metabolite production	(Sharifi et al., 2017)
*Bacillus species, Pseudomonas species, Burkholderia, Brevibacterium, Streptomyces*	Antibiotic production	(Jayaprakashvel and Mathivanan, 2011; Zhou et al., 2019)
*Pseudomonas, Bacillus, Trichoderma*	Biocontrol agents’ synthesis	(Saraf et al., 2014; Meena and Swapnil, 2019)
*Bacillus, Rhizobium, Azotobacter, Azospirillum, Frankia, Gluconacetobacter, Burkholderia, Azorhizobium, Beijerinckia, Cyanobacteria*	Biological nitrogen fixation	(Bhattacharyya and Jha, 2011; Kumar et al., 2014; Govindasamy et al., 2010)
*Pseudomonas, Bacillus, Serratia, Azospirillum, Trichoderma*	Provide Induced Systemic resistance to plants	(Choudhary et al., 2007; Nguyen et al., 2020)
*Erwinia,Serratia, Rhizobium, Mesorhizobium, Flavobacterium, Rhodococcus*	Phosphate solubilization	(Podile and Kishore, 2006; Otieno et al., 2015)
*Enterobacter* sp. PR14	ACC deaminase and antioxidant enzymes	(Sagar et al., 2020)

## 3 Microbial consortium-based approaches for PGPR activities

The PGPR activities could be influenced by various external factors for their enhanced activity. For instance, microbes could be used either in the form of monoculture or in the form of a consortium along with PGPR. This could enhance the activities performed by PGPR alone. There are several pieces of literary work thatshow the utilization of monoculture and microbial consortium for enhanced PGPR activities which of them are discussed below.

Microbial consortium (MC) carries flexibility to the oscillation of environmental factors such as temperature, pH, nutrient levels, and various toxic compounds (Pacheco et al., 2019). The diversity of metabolic pathways takes control of different members which can make it possible for the survival of consortia in fluctuating environmental conditions (Zhang and Zhang. 2022). The environmental fluctuations and metabolic diversity of the consortium are crucial to maintaining the desired functions of the PGP activities of rhizobacteria (Wang et al., 2022). For the improvement in plant growth, it is necessary to create a healthier soil environment, therefore evaluation of microbial consortium is the most important phase for their development (Kumar et al., 2019b).

### 3.1 Monoculture and consortium

In monoculture, a single strain of microorganism performs all the cell regulatory functions and a single cell carries all the burden of their metabolic pathways that leads to low efficiency and productivity of their beneficial activities (Backer et al., 2018). On the other hand, in consortia of two or more microorganisms, there is a division of labor of, therefore multiple cells in consortium exchange their levels of metabolism, energy, and function together like a cascade that results in high efficiency and productivity of their metabolic pathways (Roell et al., 2019). Monoculture suffers from the burden of the co-expression of heterologous proteins in a consortium and that results in achieving a maximum growth rate than monoculture (Liu et al., 2018). In the consortium, multiple cells are involved to perform the desired function and for that to achieve the maximum threshold of the prevailing performance of the consortium, it is required to optimize the ratio of the two cell types (Patowary et al., 2016).

**FIGURE 5:**
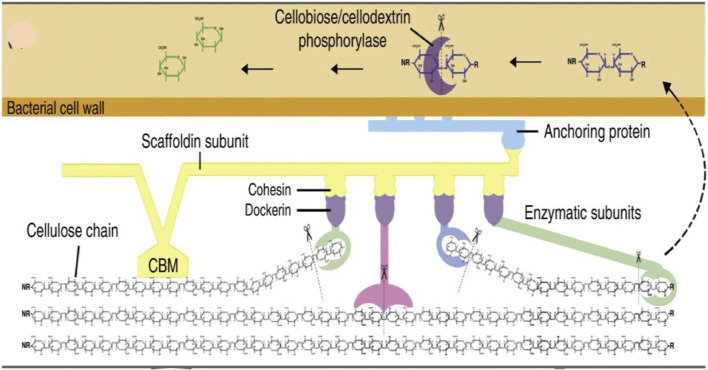
This diagram represents the cellular mechanism of lignocellulose degradation in the bacterial cell wall. Anaerobic degradation of lignocellulose is performed by a cellulosome complex which is attached to the cell wall of bacteria *via* an anchoring subunit. This complex consists of enzymes that are responsible for the hydrolysis of cellulose are and attached to a scaffolding subunit that anchors the enzymes and bacterial cells to the substrate by a carbohydrate-binding module (CBM) (Cragg et al., 2015).

**FIGURE 6:**
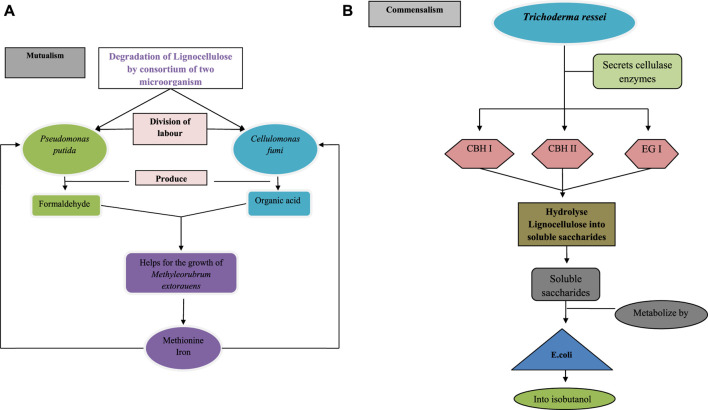
Lignocellulose degradation by **(A)** consortium of *Pseudomonas putida* and cellulomonas fungi by their mutualism interaction, division of labour between both the microorganism is present for the degradation of lignocellulose after the degradation *Pseudomonas putida* produce formaldehyde and *Cellulomonas fumi* produce organic acids, that helps in the growth of *Methyl Rubrum extorquens* which inturn secretes methionine and iron for the growth of *Pseudomonas putida* and *Cellulomonas fumi* **(B)** consortium of *Trichoderma reesei* and *Escherichia coli* by their commensalism interaction, in this interaction,*Trichoderma ressei* secretes cellobiohydrolaseI (CBH I), cellobiohydrolase II (CBH II) and endoglucanase (EG I) cellulase enzymes to hydrolyse lignocellulose into soluble saccharides and these soluble saccharides metabolize by *E. coli* into isobutanol.

For a better understanding of the benefits of the consortium over monoculture, in Figure 5 the degradation of lignocellulose is performed by a single microorganism and in Figures 6A, B by multiple microorganisms has been explained.

Examples of lignocelluloses degradation mechanisms performed by single bacteria and in the consortium (Figures 5, 6).

## 4 Rhizobacterial consortium

Various attempts have been made to find out potential consortium groups of rhizobacteria to intensify their PGP activities. Various combinations of PGPR are found in soil that is living symbiotically and performs varieties of efficient, robust activities to maintain the soil and plant health (Alves et al., 2022). The Rhizobacterial consortium also provides protection from biotic and abiotic stress to plants (Karuppiah et al., 2022). A single microorganism cannot provide protection against vast varieties of plant pathogens, that’s why there is an increasing demand for efficient consortium groups to target multiple pathogens that work as dynamic biocontrol agent (Yaduwanshi et al., 2021). Stability and compatibility between rhizobacteria are very important for their survival in nature that’s why in a rhizobacterial consortium, every single microorganism should be compatible with each other for their non-competitive establishment in the rhizosphere (El-Tarabily et al., 2021). The stability and functionality of consortia is depending upon their cooperative relationship and interactions (Che and Men. 2019). The fundamental mode of interaction between microorganisms with combinations of positive, negative, and neutral effects between two species are mutualism, commensalism, parasitism or predation, competition, amensalism, and neutralism (Yuan et al., 2021). In the consortium, rhizobacteria communicate by the exchange of metabolites and signals to coordinate their activities and to provide benefits to each other by division of labour, which permit consortia to perform a complex function and sequential processes for resource employment (Atkinson et al., 2022). In rhizobacterial consortia, the diversity of biochemical reactions enhances the utilization of resources and brings down the formation of byproducts (Zhang L. N. et al., 2019). Besides, in natural consortia, many members may metabolize harmful or toxic byproducts of primary substances like acetic acid which if not supplementary consumed will certainly waste the energy and carbon and also inhibit the production of biomass because of anion accumulation and acidification (Sun et al., 2021).

Belimov et al., 2020 developed a microbial consortium of PGPR comprising rhizobia and mycorrhizal fungi. The developed consortium applied to mutant pea species SGECd^t^which was comparable to Indian mustard in Cd tolerance and accumulation (Belimov et al., 2020).

Engineering consortiums may be an essential way to maintain the long-term stability and functionality of the rhizobacterial consortium (Han et al., 2019). The metabolic interactions have an enormous effect on the application of the rhizobacterial consortium (Woo and Pepe 2018). The metabolic compounds secreted by specific species can enhance or defeat the growth of other species and make alterations between them and also affect the function of the whole community (Berg et al., 2020). Synthetic rhizobacterial consortia are one that is created artificially by co-culturing two or more species on the basis of their compatibility (Kapoore et al., 2021). These synthetic consortia are different from natural microbial consortia in their feasible redesign of metabolic pathways to obtain convenient functions (Frioux et al., 2018). Their novel intuition will unveil the common and unique attributes of microorganisms.

### 4.1 Examples of rhizobacterial consortium

Different parameters of tomato growth have been studied by the four rhizobacterial species *Bacillus subtilis, Pseudomonas,* and the consortium, on-screen house result from the biological activity of these PGPR (Shukla et al., 2022). The phosphate solubilization ability of *Bacillus subtilis* and *Pseudomonas aeruginosa* are similar to the potent performance of *Bacillus strain* BPR7, but the consortium was magnificent in all the growth parameters. 33 isolates studied and evaluated the effect of a rhizobacterial consortium of *Bacillus spp.* on two micro-propagated bananas and concluded that bacterial consortium is the best-anticipated method to improve vegetative health and survival rates in commercial nurseries. Four isolates, *Bacillus subtilis, Pseudomonas aeruginosa, Klebsiella pneumoniae, and Citrobacter youngae* were the most effective and consistent PGPR identified (Tzirita et al., 2018; Oloyede et al., 2021).

Apart from these advantages, there are many provocations yet remain, while engineering consortium between inter and intraspecies, like their incompatibility and stabilization of their interaction to control their growth, optimize their metabolic pathways, and also understand their role in population dynamics.

## 5 Mechanism of designing synthetic rhizobacterial consortium

There are variousdrawbacks to natural consortiums because of their difficulty in culture, long operational cycle, poor stability and controllability that make a hurdle in their practical application in various fields of biotechnology (Mccully et al., 2017). The construction of a synthetic microbial consortium would be able to overcome these difficulties of the natural consortium by stabilizing their interactions and making the consortium more bearable and sustainable towards oscillating environmental conditions (Lu et al., 2020; Xu and Yu. 2021).

### 5.1 On the basis of cell-cell communication

Designing a synthetic consortium of single species by cell-cell communication is quorum sensing (QS). In QS signalling some molecules are autoinducers, they diffuse from intracellular to extracellular (Tan et al., 2020). Autoinducers trigger or coordinate the expression of certain genes when they cross a certain threshold (Almutairi 2017). OS signalling is a communication mechanism between bacteria that allow for the formation of biofilms, the production of secondary metabolites, and stress adaptation including their secretion system (Isah 2019). The secretion system plays a crucial role in cell-cell communication (Pena et al., 2019). The population density of rhizobacteria is coupled by a few special modules with the help of fluorescent protein insaptio-a temporal form of QS signalling (Mostek et al., 2020). In *E. coli* the coupling of Las/RLasL as a cell density module control with their motility control, cell density is inversely proportional to the motility (Bi and Sourjik. 2018). There is also two-way interaction by which consortium is constructed with their LasR/LasI and RhIR/RhII QS system, in this system gene expression will only respond if the population is present over a threshold cell density (Ding et al., 2018).

### 5.2 Omics tool for the synthetic assembly of consortium

Omics (Transcriptomics, trans proteomics, and metabolomics) tool that illuminates the understanding of the interaction between microorganisms in the consortium and provides precious information about functional diversity of consortium in their gene expression levels and also their metabolites profiling that helps to design and decipher the interaction of natural microbial consortium (Jia et al., 2016; Manzoni et al., 2016). Profiling of proteins and metabolites helps to artificially assemble and understand the interaction between *Ketogulonicigenium vulgare* and *Bacillus megaterium* that produce 2-keto-gluconic acid, in this interaction *B. megaterium* helps *K. vulgare* to resist reactive oxygen stress, and B*. megaterium* also provide necessary nutrients, like purine for the growth of *K. vulgare* for the production of 2-keto-gluconic acid (Wushensky et al., 2018; Yang et al., 2020).

## 6 Strategies for the selection of consortium members

For the successful construction of a synthetic rhizobacterial consortium, it is necessary to select members who are highly dependable on each other (Tabacchioni et al., 2021) to form stable relationships and embrace cross-feeding, detoxification, and biofilm formation to obtain desired functions (Castro et al., 2019; Tang et al., 2020; Vieira et al., 2021).

**FIGURE 7:**
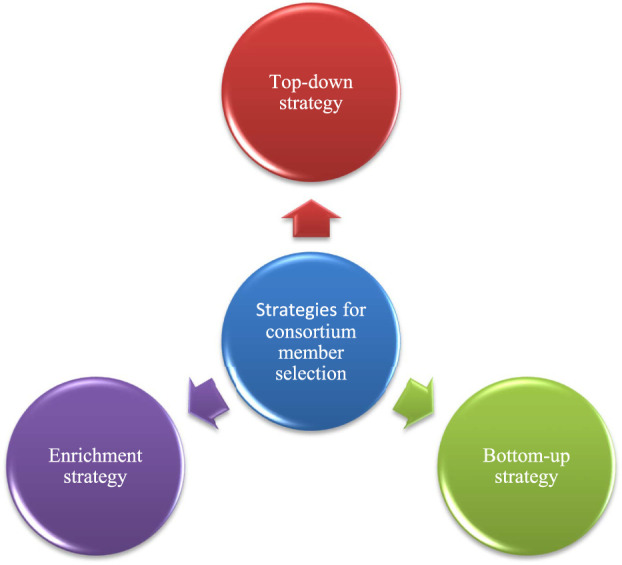
Strategies for the selection of consortium members in PGPR.

For the emergence of top-down and bottom-up strategies (San León and Nogales. 2022) a model consumer-resource model (Marsland et al., 2020) has been studied to understand the occurrence of coalescence of rhizobacteria at their community level, according to their model, collective invasions can be looked forward to producing ecological co-selection as a result of cross-feeding (Diaz-Colunga et al., 2022). There are three strategies for the selection of consortium members i.e., top-down strategy, bottom-up strategy and enrichment strategy which are shown in Figure 7.

### 6.1 Top-down strategy

This strategy occurs in natural microbial interdependence. Top-down engineering involves modifying environmental factors (pH, redox conditions, carbon source, and salt content) for assembling a natural microbial community (San León and Nogales, 2022). For the degradation of toxic chlorinated contaminants, the addition of electron donors helps in the growth of microbial consortium and also in the microbial bioremediation process (Lovley, 2022). Although, the identification of microbial culture is an important precondition for the consortium designing strategy. In spite of the fact there are various techniques, like *in situ* culture, resuscitation stimulation, and cell sorting, have encouraged research on uncultured microorganisms (Zhang X. H. et al., 2020).

A study showed that incubation time, oxygen availability, exchange in nutrient content, and spatial colocalization between members of bacterial consortium might be responsible factors to operate population dynamics in the rhizobacterial consortium (Leal et al., 2018; Santoyo et al., 2021).

An innovative top-down strategy is developed by co-cultivation of LAB (Lactic acid bacteria) and LB (lignocellulolytic bacteria) populations by using a sequential and rational modification of MRS broth by overall dilution to stimulation approach (Díaz-García et al., 2021a). In this strategy, it is very important to manipulate the whole microbial consortium to obtain the desired function.

This top-down strategy provides benefits to designing more stable and potential synthetic microbial consortia by utilizing environmental microorganisms.

### 6.2 Bottom-up strategy

For the construction of synthetic microbial consortia, this strategy is based on microbial metabolism and their interaction (Lin L. 2022). It is quite challenging to construct microbial consortia synthetically because of their complex interlinking metabolic pathways but the development of the multi-omics tool (Mallick et al., 2017; Raju and Bidlan 2017) helps to understand the genetic and molecular factors of these complex metabolic pathways, therefore it is easy to design consortium by following a bottom-up strategy and also for various quantitative models that used to uncover the dynamics of rhizobacterial consortium (Wyrwicka et al., 2019). But still, some incomplete data on genes and proteins of the metabolic network is a backbreaking challenge to engineering microbial consortia.

By using this bottom-up strategy of microbial consortium a study has been developed for the degradation of chitin (Vortmann et al., 2021). According to this study, Step-wise development of a mutualistic and non-competitive consortium in which lysine-auxotrophic *Escherichia coli substrate* converter cleaves the chitin monomer N-acetylglucosamine (GlcNAc) into glucosamine (GlcN) and acetate, but uses only acetate while leaving GlcN for growth of the lysine-secreting *Corynebacterium glutamicum* producer strain. They engineered the substrate converter strain for growth on GlcN and not for acetate. Two strains were co-cultured in the presence of a mixture of GlcN and acetate, in this mixture the growth is stabilized through the lysine cross-feeding. By using this strategy PGP activities and functions of the rhizobacterial consortium can be stabilized and optimized and make the consortium more vigorous than its natural consortium.

### 6.3 Enrichment strategy

In this strategy, there is an increase in the population of desired microorganisms by stimulating their growth and that leads to the shifting of microbial communities on the basis of their PGP activities from least potent to highly robust species (Raju and Bidlan. 2017).

### 6.4 Division of labor between members of the microbial consortium

Usually, a modified single microbial population with genetic circuits carries all the designated tasks and components in their monoculture environment, therefore single microorganisms bearing the burden of their whole metabolic pathway and the end product will form along with their ‘unprocessed’ intermediary metabolic components (Atkinson et al., 2022). Thus, there is a requirement to divide and combine the metabolic burden among members of the consortium, for the formation of the end product with their completely processed intermediary components (Czajka et al., 2017). For instance, the degradation of pyrene by a synthetic rhizobacterial consortium of three rhizobacteria (*Mycobacterium, Novosphingobium, and Ochrobactrum*) has been studied, initial degradation step of pyrene is performed by the *Mycobacterium* strain and the degradation of pyrene intermediates is efficiently performed by *Novosphingobium* and *Ochrobactrum* strains, however, *Mycobacterium* strain own the complete set of genes for pyrene degradation but these genes did not metabolize pyrene, therefore *Ochrobactrum* and *Novosphingobium* degrade intermediates (phthalate or protocatechuate) more efficiently then Mycobacterium strain (Wanapaisan et al., 2018; De Lorenzo, 2019; Singha and Pandey. 2020).

Therefore, engineering microbial consortia enable the division of labour between their members where each cell population performs one function. The use of plasmid, genome integration and spatial separation of the population is a common method to engineer these consortiums (Jia et al., 2016).

The different populations within the community may have accidental interactions and their consequences can influence the dynamics of the consortia (Gupta et al., 2020; Dos Santos et al., 2022). If the stabilization mechanism is absent among consortium members, therefore a fast-growing population can navigate the extinction of a slow-growing population (Duncker et al., 2021).

### 6.5 Steps for designing synthetic rhizobacterial consortium (SRC)

For the successful design of SRC, each step must be precise and flawless in terms of environmental sustainability. The various steps involved in designing SRC are as follows; Isolation of core microbes from the rhizosphere of plant roots, development of strategies for the selection of compatible rhizobacterial microorganisms, next-generation sequencing and network analysis, maintaining suitable culturable conditions by selecting suitable media by using the KOMODO tool (Chung et al., 2017). This is followed by synergy testing of core microbes’ population dynamics and their plant growth-promoting activities, a comparative study of cellular functions between monoculture and their consortium groups, a multi-omics tool to understand the genetic and molecular levels of metabolic pathways of consortium members, stabilization of the interaction between members of the consortium in a fluctuating environment; optimization of the metabolic pathway of interlinking consortium members. This is followed by testing of the long-term effects of the consortium, testing of SMC in consideration of the environmental impacts and finally ecological risk evaluation of SMC.

## 7 Application of natural rhizobacterial consortium and engineered consortium

### 7.1 Natural rhizobacterial consortium

In a natural ecosystem, microorganism survives in microbial communities, which organized of many interacting species, in these communities’ microbes exchange their metabolites, cross-feed and plays important role in the global cycling of gases and these natural consortia uses for decades for various purpose (Ben Said and Or, 2017).

An example of a natural consortium is two coexisting bioleaching bacteria called Ferroplasma acidiphilum and Leptopirillum ferrriphilum (Merino et al., 2016), their symbiotic association helps in oxidizing sulfur and iron consisting minerals. The natural reservoir of microbial and fungal consortia is herbivore guts, these consortia work synergistically to release a manifold range of cellulolytic enzymes for the degradation of plant biomass (Xu and Yu 2021).

The natural consortium is not stable in fluctuating environmental conditions because of their incompatibility, metabolic burden, and less productivity of biomolecules and products. Microbial synthetic biology helps to engineer rhizobacterial consortium, to accomplish all the drawbacks of natural consortium and enhance productivity.

### 7.2 Applications of engineered rhizobacterial consortium (ERC)

Designing synthetic rhizobacterial consortium in concern with an environmental ecology, that promotes the beneficial activities of PGPR for a long time and stabilizes their interactions, leads to revealing microbial ecosystem, and for those various studies have been conducted in the last decade to obtain benefits from the vigorous multifunctional rhizobacterial consortium. Here we are compiling all the applications of formulated rhizobacterial consortium (Table 2).

**TABLE 2:** Engineered rhizobacterial consortium and their applications in environmental ecology and biotechnology.

S. No	Rhizobacterial consortium between	Benefits in environmental ecology and biotechnology	References
1.	*Trichormus variabilis with Acinetobacter, Exiguobacterium and Pseudomonas spp.*	Recycle and save water in household appliances	(Congestri et al., 2020)
2.	*Mycobacterium* spp. PO1 and PO2, *Novosphingobium pentaromativorans* PY1, *Ochrobactrum* sp. PW1, and *Bacillus* sp. FW1	Synergistic degradation of pyrene in the consortium will facilitate the application of the defined bacterial consortium in the process of bioremediation.	(Wanapaisan et al., 2018)
3.	*Saccharomyces cerevisiae, Salmonella enterica, Klebsiella pneumoniae,* and *E. coli*	Costless production of useful metabolites for plants	(Pacheco et al., 2019)
4.	*Zobellella taiwanensis AT1–3*, and *Bacillus pumilus* HKG212	textile effluent degradation	(Das and Mishra 2017)
5.	*Pseudomonas* sp. and *Paenibacillus* sp.	improve saccharification (i.e., the release of sugars from agricultural plant residues) processes in biorefineries.	(Díaz-García et al., 2021b)
6.	*Clostridium thermocellum* and *Methanobacterium thermoautotrophicum*	produce hydrogen gas, methane, acetic acid, and ethanol	Weimer and Zeikus (1977)
7.	*Enterobacter hormaechei* (AM122) and *Lysinibacillus xylanilyticus* (DB25)	plant growth, yield, and aroma enhancement in basmati and non-basmati rice varieties.	(Dhondge et al., 2021)
8.	*Bacillus pumilus KS2 and Bacillus cereus R2*	For decontamination of the sites which are contaminated with toxic pollutants of crude oil containing PAHs.	(Patowary et al., 2016)
9.	*Burkholderia ubonesis* la3c3, *Burkholderia vietnamiensis* la4 and *Citrobacter bitternisp* 9a3m	Reducing the application of nitrogen fertilizers	(Ríos-Ruiz et al., 2020)
10.	*Bacillus sp*. AC-225 *Serratia sp*. AD-7 and *Bacillus sp.* AC225	improves wheat growth in Chilean Andisols under water shortage conditions	Inostroza et al., 2017
11.	*Bacillus thuringiensis* strain RBI 2AB1.1, *Cyanobacteria* RZ2AB2.1., *Bacillus subtilis* BSn5 RBI IPBL 2.3 and *Bacillus cereus strain* APSB03 RBI 2AB 2.2,	Enhance the growth of tomato plants and protection from the plant pathogen	(Yanti et al., 2021)
12.	*Streptomyces sp*.X52 *Peribacillus sp*. P10 and *Pseudomonas sp*. P8,	Improving the salt resistance of crop	(Peng et al., 2021)
13.	*Pseudomonas* spp. DPC12, *Ochrobactrum anthropi* DPC9, *Acromobacter* spp. PSA7, PSB8, DPB13, DPB15, DPB16, and *Variovorax paradoxus* RAA3	reduce water stress in wheat (*Triticum aestivumL.*) plant	(Chandra et al., 2019b)
14.	*Pseudomonas putida* IIHR-PP17 and nematophagous fungi *Trichoderma viride* IIHR TV-2	Enhancement of De-oiled neem cake to provide protection from nematodes in gherkin fruit plants	(Kamalnath et al., 2019)
15.	*Bacillus* sp Cr and *Pseudomonas* sp. Crb	Biofertilizer of the soybean plant	Meliha et al. (2013)
16.	*Serratia marcescens* KAHN 15.12 *Burkholderia cepacia* KD 2.10, and *Bacillus thuringiensis*SAHA 12.12	Biofertilizer for chilli plants	(Swandi et al., 2019)
17.	*Bacillus megaterium, Pseudomonas aeruginosa Arthrobacter Enterobacter sp.,* and *Chlorophenolicus*	For the improvement of wheat plants’ health	Kumar et al. (2020)
18.	Four *Pseudomonas* strains	Protection from weeds in wheat plants	Raza et al. (2021)
19.	*Bacillus* and *Pseudomonas* group	Inhibition of nematode pathogens in the coffee plant	(Asyiah et al., 2020)
20.	Rhizobacterial consortium	Promotes the growth of *Camellia sinensis* (Tea plant)	(Shang and Liu 2020)
21.	*Bacillus megaterium* CAM12 and *Pantoea agglomerans* CAH6	Inhibition of aluminium and drought stress and improving the growth of Mung bean	(Silambarasan et al., 2019)
22.	*Bacillus subtilis* SM21 *Bacillus cereus* AR156, and *Serratia sp.* XY21 is called BBS.	Biocontrol agent for sweet pepper	(Zhang et al., 2019a)
23.	PGPR strains (*Acinetobacter sp*. BS17 plus *RahnellaAquatilis sp*. PGP27) and two rhizobia (*Ensifer meliloti* sp. RhOF4 and *Ensifer meliloti* sp. RhOF155).	For the growth of *Vicia faba* L. and *Triticum durum* plant	(Raklami et al., 2019)
24.	*Bacillus subtilis* and *Bacillus pumilus*	Enhancement of fruit yield of *Capsicum annum*	(Kaushal et al., 2019)
25.	Rhizobium (M1, LSMR1, and LSMR2 varieties) is combined with rhizobacteria (LSRB1, DBRB2, and LSRB3)	Improvement of the growth of mungbean seeds	(Kumari et al., 2020)
26.	*Burkholderia gladioli*, *Pseudomonas sp*. and *Bacillus* subtilis.	Improvement of the growth of fenugreek plants	(Kumar et al., 2020)
27.	*Bacillus velezensis* Vb1, *B. paramycoides* Vb6., and *B. para mycoides* Vb3	biocontrol agent for faba bean roots	(El-Sersawy et al., 2021)
28.	Nitrogen-fixing *Azospirillum sp*. and phosphate-solubilizing bacteria	replacement of biofertilizers in tea nurseries	Teenakoon et al., 2021
29.	*Enterobacter cloacae* RCB980 (A3), *Klebsiella pneumonia kpa (A4),* and *Klebsiella sp*XT-2 (A7)	in remediation of cadmium-contaminated soil	(Yankey et al., 2021)
30.	Diesel degrading rhizobacterial consortium	Rhizoremediation of polyhydrocarbons	(Eze et al., 2021)
31.	*Lactobacillus* sp., *Pseudomonas fluorescens Azotobacte*r sp., *Bacillus subtilis*, *Actinomyces sp*., *Bacillus polymyxa*, and *Rhizobium*	Increase the weight of mulberry leaves	(Mustaka et al., 2022)
32.	R011 isolate + R08 isolate + *Rhizobium* sp. LM-5.*Rhizobium* sp. LM-5, R08 isolate, R011 isolate, R08 isolate + *Rhizobium* sp. LM-5, R011 isolate + R08 isolate, LM-5, R011 isolate + *Rhizobium* sp.	Restoration of biological fertility and soil health	(Purwanto and Suharti 2021)
33.	*Bacillus subtilis, Klebsiella pneumoniae, Pseudomonas aeruginosa, and Citrobacter youngae*.	Growth enhancement of Tomato plant	(Oluwambe 2016)
34.	*Pseudomonas sp, Acinetobacter with Trichormus variabilis*	For the treatment of wastewater	(Congestri et al., 2020)

#### 7.2.1 Rhizobacterial consortium as a biocontrol agent for plants

Biological control of plant disease is defeating plant pathogens by using varieties of biological agents such as plant metabolites, anti-pathogenic microorganisms, root exudates, synthetic fertilizers *etc.* Similarly, the rhizobacterial consortium is used as a biological control agent for plants and improves plant productivity.

The study shows rhizobacterial consortia from arid ecosystems improve wheat growth in Chilean Andisols under water shortage conditions and reduce water stress by ACC deaminase-producing rhizobacteria consortium (Inostroza et al., 2017; Chandra et al., 2019a). Many studies enlighten that rhizobacterial consortium is a biocontrol agent and provide anti pathogenicity for tomato and wheat plants four *Pseudomonas* strains were selected for the formulation of consortium on the basis of their efficient PGP activities and their combination with sorghum allelopathic water extract was found to be more effective to control *Phalaris minor* Retz. and *Avenafatu* L. weed of wheat plants for sustainable production (Raza et al., 2021), Consortium of *Pseudomonas putida* IIHR-PP17 and nematophagous fungi *Trichoderma viride* IIHR TV-2 were used to enrich the de-oiled neem cake for the protection from *Meloidogyne incognita* and *Fusarium oxysporum* f sp. in gherkin (*Cucumis anguria* L.) fruit plant (Kamalnath et al., 2019). The consortium of *Bacillus thuringiensis, Bacillus cereus, Bacillus subtilis* strains, and *cyanobacteria* have the best synergistic ability that helps to increase the growth of tomato plants and provide protection from plant pathogens (Yanti et al., 2021). Tetra combination of consortium used for the wheat growth (Kumar et al., 2020) and also the consortium of *Bacillus* and *Pseudomonas* group that efficiently inhibit the growth of *Pratylenchus coffee* and enhance the growth of *Robusta coffee* (Asyiah et al., 2020). Consortium against *Fusarium oxysporum* helps to provide protection from wilt disease to faba bean plant roots (El-Sersawy et al., 2021). Three PGPR strains *Bacillus cereus* AR156, *Bacillus subtilis* SM21 and *Serratia sp.* XY21 called BBS treatment in the soil leads to the shifting of the microbial community that suppressed soil-borne disease and improves the soil’s chemical properties (Zhang L. et al., 2019).

#### 7.2.2 Rhizobacterial consortium for stress tolerance in plants

**FIGURE 8:**
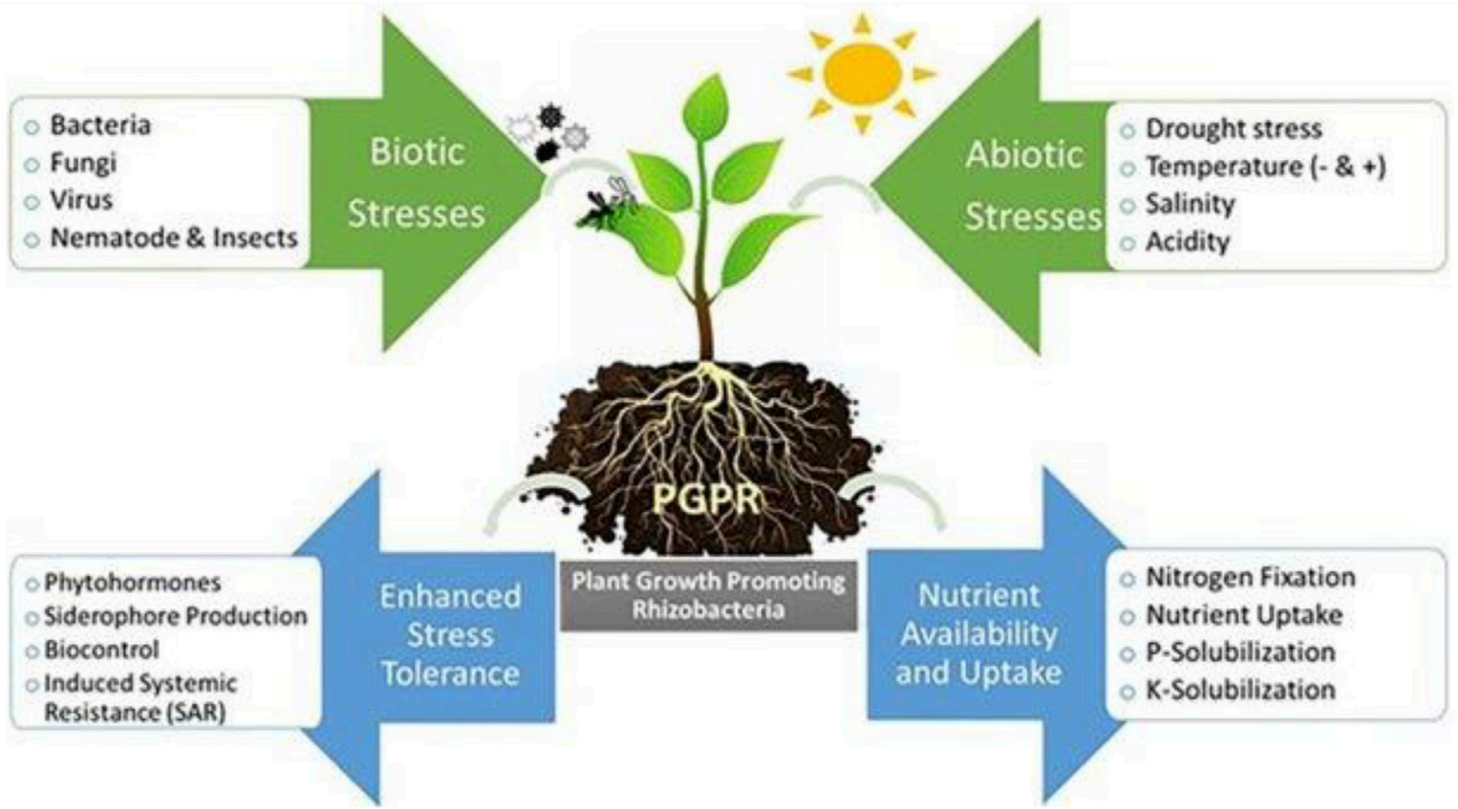
PGPR and the interaction of plants in the rhizosphere (Shah et al., 2021b).

Plants acclimatize their physiology and morphology to thrive in high-stress environmental conditions. The different rhizobacterial consortiums are designed to bring about more plant tolerance against biotic and abiotic stresses. Figure 8 shows PGPR and the interaction of plants in the rhizosphere.

PGPR consortium improves salt tolerance in maize plants by using the consortium of *sp*. P8, *Paenibacillus sp*. P10 and *streptomyces sp*. X52 (Cavalu and Damian, 2003; Peng et al., 2021). The consortium of *Bacillus* sp Cr and *Pseudomonas* sp. Crb shows a significant increase in soybean plant growth and mineral uptake (Meliha et al., 2013) and treatment of Zn malnutrition in staple rice grains by the formulation of the rhizobacterial consortium (Vaid et al., 2020). *Bacillus megaterium* CAM12 and *Pantoea agglomerans* CAH6 were selected on the basis of their ability to tolerate high Al (8 mM) and drought stress. By using this consortium *Vigna radiata* plants can tolerate more abiotic stresses and efficiently grow in high levels of Al soil (Silambarasan et al., 2019). Two studies show the enhancement of yield and aroma of basmati and non-basmati rice by using *Enterobacter hormaechei* (AM122) and *Lysinibacillus xylanilyticus* (DB25) (Dhondge et al., 2021). *Saccharomyces cerevisiae, Salmonella enterica, Klebsiella pneumoniae, and E. coli,* members of the consortium frequently release and exchange beneficial metabolites that are used by plants (Pacheco et al., 2019).

#### 7.2.3 Rhizobacterial consortium for bioremediation

Two studies show the engineering of rhizobacterial consortium for the treatment of contaminated soil and wastewater (Yankey et al., 2021) (Congestri et al., 2020) a study is conducted for the metagenomic analysis of PGPR consortium members to uncover the varieties of diesel degrading rhizobacterial consortium and their genetic variation (Eze et al., 2021). The degradation of pyrene by the synergistically rhizobacterial consortium has been studied (Cavalu and Damian, 2003; Wanapaisan et al., 2018).

#### 7.2.4 Rhizobacterial consortium as a biofertilizer

Varieties of synthetic fertilizers are used simultaneously to maintain the nutrient balance for plants but these synthetic fertilizers also cause harmful effects on our soil microbiome, therefore engineered rhizobacterial consortium can also be used as a biofertilizer tool. Many studies have been conducted to provide natural fertilizers, various phosphate solubilizing rhizobacteria and *Bradyrhizobium japonicum* as a biofertilizer in a soybean plant (Meliha et al., 2013), reducing the application of nitrogen fertilizers by using rhizobacterial consortium (Ríos-Ruiz et al., 2020).

A study shows the growth enhancement of tomato plants by the cooperative PGP activities in the consortium (Oluwambe 2016; Ríos-Ruiz et al., 2020) and the restoration of biological fertility and soil health (Purwanto and Suharti 2021). The consortium of *Lactobacillus* sp., *Bacillus subtilis*, *Actinomyces sp*., *Azotobacte*r sp., *Bacillus polymyxa*, *Pseudomonas fluorescens*, and *Rhizobium Has* been studied on the basis of their PGP activities that help to increase the weight of mulberry leaves (*Morus indica*) than single rhizobacteria (Mustaka et al., 2022). Isolation of nitrogen-fixing *Azospirillum sp*. and phosphate solubilizing bacteria from the three-soil series of Sri Lanka was used as a consortium to fix the nutrient uptake in tea plants and as biofertilizers (Tennakoon et al., 2021). Rhizobacterial consortiums are used as biofertilizers to increase the growth of chilli plants (Swandi et al., 2019). Microbial consortium TCM was selected in a study for field trial and showed that TCM *consortium improves* the effectiveness of rhizospheric nutrition and promotes the growth of Camellia sinensis (Shang and Liu 2020). Fenugreek plant and its yield is significantly increased in consortium than their single inoculation (Patowary et al., 2016; Kumar et al., 2020). In a study, mung bean seeds (SML668 and SML832) were inoculated with single rhizobium and in combination with rhizobacteria. The combination of rhizobacteria and rhizobium improves mungbean overall growth as compared to their single inoculation with rhizobium (Kumari et al., 2020). A consortium of two PGPR isolates (*Bacillus subtilis* and *Bacillus pumilus*) was used by the consortium to increase the yield of the bell pepper plant and disease resistance under field conditions (Kaushal et al., 2019). Two PGPR strains (*Acinetobacter sp*. BS17 and *Rahnella Aquatilis* sp. PGP27) and two rhizobia (*Ensifer Meliloti* sp. RhOF4 and *Ensifer Meliloti* sp. RhOF155) were used to improve the growth parameters of plants. The experimental results show that rhizobacterial and mycorrhizal consortiums appear to adapt to the soil’s native microflora when applied in the field for the growth of *Vici faba* L. and *Triticum durum* (Raklami et al., 2019).

All the studies of engineering rhizobacterial consortium are based on their PGP activities that help to improve plants’ growth, productivity, their anti-pathogenicity, makes them tolerant against biotic and abiotic stresses, and helps in the degradation of various Polycyclic Aromatic Hydrocarbons (PAHs) compounds. Synthesis of the rhizobacterial consortium is a multistep process from the selection of the members to their implications in the field, that’s why in all the studies we found a common method of designing experiments which is a comparison of PGP activities between single rhizobacteria and with their consortium and that establish the functional dominance of rhizobacterial consortium over the single rhizobacteria.

## 8 Future prospects

All the field experimental studies show, that the rhizobacterial activities in the consortium are more robust and dynamic than single rhizobacterial activities but it is necessary to perceive the “long-term consortium effect and stability” while performing in field trials and for that, existing studies must be practised more in the field and diversified studies should be conducted to promote understanding of consortium mechanisms. Therefore, the studies suggested that there is an urgent requirement to design and establish the rhizobacterial consortium *in vitro* to find out their communication mechanism and confirmations are required that consortium groups can be applied in the field for plant growth promotion, soil health, and our environmental ecology. A lot of research-based experiments are required in this field involving microbial consortiums. Moreover, we need to test their findings more in field trials for many years to establish the very long-term effect of consortium in all environmental conditions.

## 9 Conclusion

The rhizosphere region is a highly active zone which harbours various important microbes. The release of root and plant exudates which are rich in nutrients is the major factor in the microbial activities in the rhizosphere region. PGPR has become important for almost all activities today including sustainable agriculture, biofertilizers *etc.* The utilization of microbial consortium in PGPR has shown better adaptation of consortium groups in the natural environment. The microbial consortium-based PGPR showed significant activities that refine and upgrade the health and productivity of plants. Experimental evidence proved that rhizobacteria perform more beneficial activities in their consortium than single rhizobacteria. Thereare still some areas to be revealed for the development of the consortium. The most important challenge to designing consortiums is to understand the complexity of microbial interactions in the natural environment, therefore while designing consortia it will become uncomplicated to understand the metabolic pathways, compatibility between microorganisms and their limitations. Another challenge while designing a consortium is the exposure of mutants. Minimizing the mutants is the utmost priority to be fulfilled. Due to the breathtaking robust applications of the rhizobacterial consortium, in the future, furthermore, studies must be conducted to design and discover more beneficial rhizobacterial consortium groups and also to make awareness among farmers to utilize its applications for sustainable environment and human wellbeing.

## Abbreviations

PGPR, plant growth promoting rhizobacteria; MC, microbial consortium; RC, rhizobacterial consortium; SRC, synthetic rhizobacterial consortium; ERC, engineered rhizobacterial consortium; ACC, 1-aminocyclopropane 1-carboxylate deaminase; IAA, indole acetic acid; PSB, phosphate solubilizing bacteria.
